# Dependency relationships between IFT-dependent flagellum elongation and cell morphogenesis in *Leishmania*

**DOI:** 10.1098/rsob.180124

**Published:** 2018-11-21

**Authors:** Jack Daniel Sunter, Flavia Moreira-Leite, Keith Gull

**Affiliations:** 1 Department of Biological and Medical Sciences, Oxford Brookes University, Gipsy Lane, Oxford OX3 0BP, UK; 2 Sir William Dunn School of Pathology, University of Oxford, South Parks Road, Oxford OX1 3RE, UK

**Keywords:** IFT, *Leishmania*, morphogenesis, amastigote, differentiation

## Abstract

Flagella have multiple functions that are associated with different axonemal structures. Motile flagella typically have a 9 + 2 arrangement of microtubules, whereas sensory flagella normally have a 9 + 0 arrangement. *Leishmania* exhibits both of these flagellum forms and differentiation between these two flagellum forms is associated with cytoskeletal and cell shape changes. We disrupted flagellum elongation in *Leishmania* by deleting the intraflagellar transport (IFT) protein IFT140 and examined the effects on cell morphogenesis. *Δift140* cells have no external flagellum, having only a very short flagellum within the flagellar pocket. This short flagellum had a collapsed 9 + 0 (9v) axoneme configuration reminiscent of that in the amastigote and was not attached to the pocket membrane. Although amastigote-like changes occurred in the flagellar cytoskeleton, the cytoskeletal structures of *Δift140* cells retained their promastigote configurations, as examined by fluorescence microscopy of tagged proteins and serial electron tomography. Thus, *Leishmania* promastigote cell morphogenesis does not depend on the formation of a long flagellum attached at the neck. Furthermore, our data show that disruption of the IFT system is sufficient to produce a switch from the 9 + 2 to the collapsed 9 + 0 (9v) axonemal structure, echoing the process that occurs during the promastigote to amastigote differentiation.

## 1. Introduction

*Leishmania* are eukaryotic protozoan parasites that cause the leishmaniases, a set of neglected tropical diseases that affect millions worldwide [1]. The parasites have a complex life cycle in which they alternate between an insect vector and a mammalian host, while adopting different morphologies. *Leishmania* has two major cell morphologies: the promastigote found in the sand fly vector, which is associated with an extracellular lifestyle; and the amastigote in the mammalian host, associated with intracellular proliferation within macrophages. Promastigotes have an elongated cell body with a long motile flagellum that has a 9 + 2 arrangement of microtubules in the axoneme, enabling the parasite to traverse through the sand fly digestive tract [2]. Conversely, amastigotes have a more spherical cell shape with a short, immotile flagellum with a collapsed 9 + 0 (9v) axonemal structure that does not extend beyond the cell body.

Despite these different morphologies, the overall organization of the *Leishmania* cell follows a conserved pattern found within the Kinetoplastida, which includes other parasites such as *Trypanosoma brucei*. While the shape of the *Leishmania* cell is defined by an array of regularly spaced microtubules that run below the plasma membrane, the cytoplasmic architecture converges on the basal body of the flagellum [3–7]. The basal body is physically linked to the single branched mitochondrion via a tripartite attachment complex that connects the basal body to the mitochondrial DNA complex (the ‘kinetoplast’) [8,9]. In addition, a flagellum extends from the basal body that emerges from the cell at the anterior end.

At the base of the flagellum is an invagination called the flagellar pocket, which is the only site of exo- and endocytosis in the cell [4,10,11]. The *Leishmania* flagellar pocket has two defined regions: a bulbous region of approximately 1 µm in length immediately anterior to the basal body; and the flagellar pocket neck region, where the flagellar pocket and flagellum membranes are closely apposed for a distance of approximately 1 µm, until the flagellum emerges from the cell at the anterior end [11]. At the proximal end of the neck, two distinctive filaments encircle the flagellar pocket membrane in an oblique C-shaped path, defining the flagellar pocket collar, a constriction that marks the limit between the bulbous and the neck regions of the pocket [11].

In *Leishmania*, the flagellum is attached to the cell body within the flagellar pocket neck region via a complex cytoskeletal structure termed the flagellum attachment zone (FAZ). Our group has previously identified components of these cytoskeletal and attachment structures and described the complex three-dimensional organization of the *Leishmania* FAZ, both in promastigotes and in amastigotes [11]. Underlying the neck membrane in the cell body side of the FAZ, a number of electron-dense structures are found with a defined organization. The typical microtubule quartet (MtQ) that emerges from the basal body region performs a helical path around the pocket bulbous region, passing through a gap in the path of the collar filaments, and then running below the neck membrane. A row of electron-dense complexes and a broad FAZ filament are always found next to the MtQ in the neck. Along the line of flagellum attachment, there is a distinctive row of junctional complexes; however, beneath the majority of the flagellar pocket neck membrane, there is a band of distributed electron density.

During the promastigote to amastigote differentiation, in addition to the dramatic shortening of the flagellum and its conversion to a 9 + 0 configuration, the organization and shape of the flagellar pocket changes [11,12]. The flagellar pocket neck region contracts around the flagellum, reducing the distance between the flagellum and flagellar pocket neck membranes. Moreover, the distal end of the neck constricts tightly around the flagellum as it exits the amastigote cell body. These changes in flagellar pocket shape are accompanied by concomitant changes in the organization of the FAZ and its constituent proteins [11].

In *T. brucei*, two factors make the basal body a central organizer of cell morphogenesis: first its link to the mitochondrion, which means that the basal body is essential for mitochondrial genome segregation [13]; and, second, its association with the FAZ, the lateral attachment of the flagellum to the cell body. Both cytoplasmic and flagellum components interact to form the FAZ, and correct FAZ formation is important for accurate cytokinesis furrow positioning during cell division [14–16]. Consequently, *T. brucei* cells without a flagellum have a shorter FAZ and a shorter cell body [17,18] and are not viable, due to the catastrophic effects of the lack of a flagellum in the hierarchy of cell morphogenesis.

The dependency relationships between cell cycle events, basal body/flagellum-linked structures and morphogenesis in *T. brucei* were established via the use of DNA synthesis inhibitors, anti-microtubule agents and the production of mutants defective in flagellum assembly [17–21]. The flagellum assembly mutants were generated by perturbing intraflagellar transport (IFT), an evolutionarily conserved machinery responsible for the assembly of eukaryotic cilia and flagella (reviewed in [22]). IFT is the major mechanism for construction, maintenance and turnover of the flagellum and the multiple components involved form two complexes—A and B [23–25]. While complex B proteins are involved in the kinesin-based ‘anterograde’ transport of material to the flagellum tip, complex A proteins are involved in the dynein-based ‘retrograde’ transport of proteins toward the basal body, recycling material from the flagellum tip to its base. Comparatively less is known about the IFT-A than about IFT-B; however, evidence from different systems indicates that the IFT-A components IFT140, IFT122 and IFT144 interact to form a sub-complex that is likely to represent the core of IFT-A and are required for correct retrograde IFT function [23,26,27]. Interestingly, ablation of IFT-A components often results in the formation of a bulge at the tip of the flagellum caused by an accumulation of IFT-B complex proteins that failed to return to the base of the flagellum for recycling [28–31].

While the flagellum is clearly a key unit of kinetoplastid cell architecture, previous studies have shown that, unlike in *T. brucei*, the elongated flagellum is not required in *Leishmania mexicana* for continued cell growth in culture [32]. This study showed that the deletion of the cytoplasmic dynein *LmxDHC2.2*, which is likely to be the IFT retrograde dynein motor, resulted in promastigote cells with very short flagella that were not visible by light microscopy and restricted to the flagellar pocket. This is at first sight a rather intriguing result; however, the Beverley group ([33], S. Beverley 2017, personal communication) has recently repeated a similar result with an *IFT140* deletion in *L. donovani.* Moreover, Zauli and co-workers [34] isolated a naturally occurring *Leishmania* mutant that has a very short flagellum extending just beyond the flagellar pocket. These results raise the issue of what dependency relationships are involved in flagellum initiation and extension in *Leishmania* and what the precise phenotype is of the resulting flagellum and cells.

Here, we used *L. mexicana* cells with a strong flagellum elongation defect—due to the lack of the IFT component IFT140—to investigate the dependency relationship between flagellum elongation and the construction of different structures in the cell, thereby defining the position of flagellum assembly in the hierarchy of cell morphogenesis events in *Leishmania*.

## 2. Results

### 2.1. Deletion of *IFT140* results in shorter cells with no external flagellum

**Figure 1.**
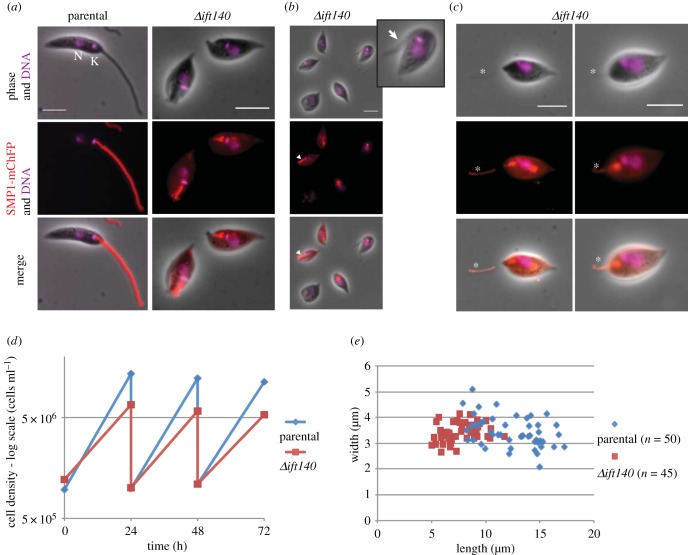
*Δift140* cells have no visible flagellum, are smaller and grow at a slower rate than the parental cells. (*a–c*) Micrographs of parental and *Δift140* cells with the SMP1::mChFP flagellar membrane marker in red and the DNA stain (Hoechst) in magenta. Nucleus (N) and kinetoplast (K) are highlighted in the parental cell image. Note the anterior positioning of the kinetoplast in *Δift140* cells (as in parental cells), despite the lack of an external flagellum. A cytoplast shearing off from a cell is indicated by a white arrow in the zoomed inset (*b*), and a short linear SMP1::mChFP signal is indicated by a white arrowhead (*b*). Plasmanemes labelled with SMP1::mChFP emerged from the anterior end of *Δift140* cells (asterisk in (*c*)). By phase contrast, these plasmanemes are clearly different from flagella (which appear thicker and more regular). *Δift140* cells have different intensities of SMP1::mChFP signal (*a–c*), and some cells have SMP1::mChFP in the cell membrane (*c*). Scale bars = 5 µm. (*d*) Growth curve of parental and *Δift140* cells. The *Δift140* cells grow consistently slower than the parental cells. (*e*) Cell body length and width of 1K1N cells from parental and *Δift140* populations. *Δift140* 1K1N cells are shorter than the parental 1K1N cells.

To test whether the morphogenesis of a range of cytoskeletal structures and positioning of organelles was dependent on flagellum assembly, we generated an *IFT140* deletion cell line by sequential replacement of the two alleles of the *IFT140* gene with resistance markers. To confirm the deletion was successful, we tested both the integration of the resistance markers into the correct locus and the loss of the *IFT140* coding sequence by PCR (electronic supplementary material, figure S1). Promastigote cells in which *IFT140* was deleted had no observable external flagellum by light microscopy (figure 1*a*). However, they were proliferative in culture (figure 1*d*), and we had no difficulty in obtaining and maintaining these mutant cells.

The *Δift140* mutant was generated in a cell line that expresses a fluorescently tagged protein, SMP1::mChFP. SMP1 localizes to the flagellum and associates with the membrane via fatty acid modification of its N-terminus and is therefore a convenient marker for the flagellum membrane [12,35]. In parental cells, tagged SMP1 was found along the membrane of the flagellum, from its base near the kinetoplast to the flagellum tip, but was not found on the membrane of the flagellar pocket (figure 1*a*) [12]. In the *Δift140* cells, we observed a range of SMP1 signals, from cells with a barely discernible SMP1::mChFP signal (approx. 34%, *n* = 67) to those that had a short linear signal at the anterior end of the cell that looked remarkably like a short flagellum, within the flagellar pocket (approx. 66%, *n* = 67; figure 1*b*). Unlike the parental cells, approximately 16% (*n* = 67) of the *Δift140* cells had an SMP1::mChFP signal on the cell body membrane in addition to signal from the flagellar pocket (figure 1*c*). Also, for approximately a third (22/67) of *Δift140* cells, SMP1::mChFP-positive material was observed emerging from the flagellar pocket at the anterior end of the cell (figure 1*c*). In the corresponding position on the phase image, a thin tubular structure was observed that appeared clearly distinct from a flagellum (compare the phase contrast image of the flagellum in figure 1*a* with the thin tubule marked with an asterisk in figure 1*c*). We termed these structures ‘plasmanemes’ based on their similarity to the previously described structures observed in trypanosomes and *Leishmania* [36,37]. While plasmanemes were labelled with SMP1::mChFP, indicating that they represent flagellar membrane protrusions, they lacked an internal flagellar cytoskeleton by electron microscopy and an anti-PFR antibody did not detect a PFR (electronic supplementary material, figure S2). In addition, we also observed SMP1::mChFP labelled plasmanemes by light microscopy as distinct structures not associated with any cells (electronic supplementary material, figure S2).

The *Δift140* cells were able to grow in culture, albeit at a slower rate than the parental cell line (figure 1*d*). The reduced growth rate may in part be due to the undoubted errors in cell division—such as the generation of cytoplasts (figure 1*b*)—that we observed by light microscopy. In addition to the lack of an external flagellum, the *Δift140* promastigote cells appeared considerably shorter than the parental cells. To quantify this phenotype, we measured cell length at a specific cell cycle stage (G1/early S), for both *Δift140* and parental cell lines (figure 1*e*). The kinetoplast and nucleus duplicate and divide at a consistent point within the cell cycle and, therefore, one can place *Leishmania* cells in a specific cell cycle stage based on the number of kinetoplasts and nuclei. The *Δift140* cells with the ‘1K1N’ configuration (i.e. in the G1/early S stage) had a length of 7.6 ± 1.6 µm compared with the parental cell length of 12.5 ± 2.6 µm (figure 1*e*). The range of lengths of the parental cells (7.9–17.2 µm) was much greater than that of the *Δift140* cells (5.0–11.2 µm).

Despite the lack of an external flagellum and the decrease in cell length, the overall position of the nucleus and kinetoplast within 1K1N *Δift140* cells appeared normal, with the kinetoplast anterior to the nucleus as in parental cells (figure 1*a*). Moreover, the kinetoplast of *Δift140* 1K1N cells appeared to be located at a similar distance from the anterior end of the cell.

### 2.2. *IFT140* deletion causes redistribution of the IFT-B complex protein IFT52

**Figure 2.**
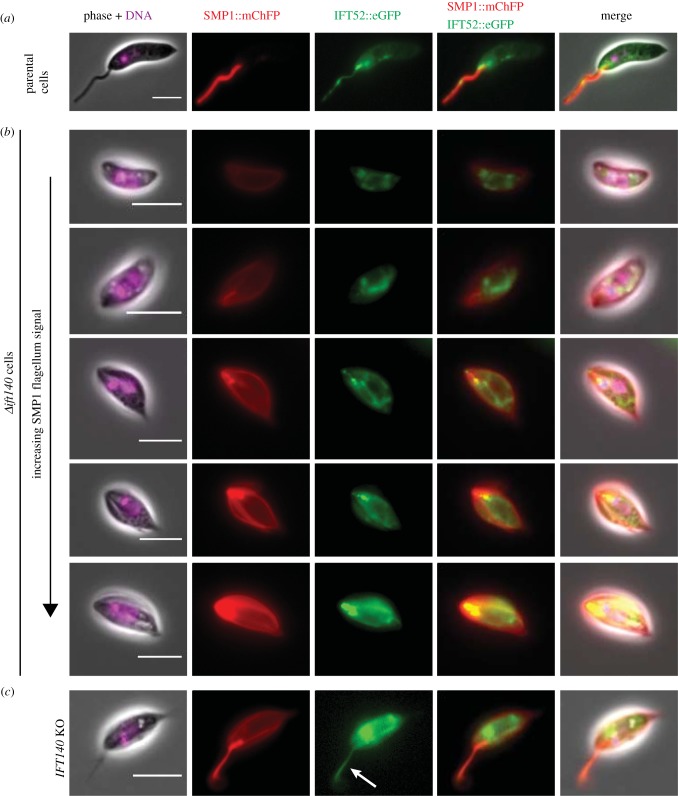
*IFT140* deletion causes redistribution of the IFT-B complex protein IFT52. Micrographs of parental (*a*) and *Δift140* cells (*b,c*) expressing IFT52::eGFP, with DNA stain (Hoechst) in magenta, SMP1::mChFP in red and IFT52::eGFP in green. (*a*) In parental cells, IFT52::eGFP is distributed in spots along the flagellum with a bright spot at the base of the flagellum. (*b*) In *Δift140* cells IFT52::eGFP is found within the cytoplasm with a variable amount associated with the short flagellum, as identified by the SMP1::mChFP signal in the flagellar pocket area. (*c*) In some cells IFT52::eGFP is associated with SMP1::mChFP-positive plasmanemes (white arrow) that emerged from the anterior end of *Δift140* cells (and were clearly distinct from external flagella by phase contrast). Scale bars = 5 µm.

To investigate the effect of *IFT140* deletion on IFT-B complex function, we tagged IFT52, an IFT-B complex protein, with eGFP in *Δift140* cells (figure 2). In parental cells, IFT52::eGFP had the expected distribution, with a strong signal at the base of the flagellum and spots along the flagellum (figure 2*a*). Conversely, in *Δift140* cells, the IFT52::eGFP localization was different, with a relatively strong signal in the cytoplasm and variable levels of IFT52::eGFP associated with the flagellum region (figure 2*b*). The amount of IFT52::eGFP in the anterior end of the cell appeared proportional to the amount of SMP1::mChFP, with more IFT52::eGFP observed when the SMP1::mChFP signal was stronger (figure 2*b*). When the IFT52::eGFP was associated with the short flagellum inside the flagellar pocket (as identified by a stronger and linear SMP1::mChFP signal at the anterior end of the cell), we observed distinct spots likely to correspond to the base and tip of the short residual flagellum (figure 2*b*); however, the spot at the tip was only observed in approximately 8% (*n* = 173) of cells. This IFT52::eGFP localization suggests that, in some cells at least, *IFT140* deletion led to the reduced retrograde movement of IFT-B complex proteins from the tip to the base, leading to an accumulation of IFT52 at the flagellum tip. Membrane plasmanemes were again discernible in images of *Δift140* cells and, interestingly, IFT52::eGFP was found in these SMP1::mChFP-positive plasmanemes (figure 2*c*), supporting the hypothesis that these plasmanemes are derived from flagellum.

### 2.3. *Δift140* promastigotes have an amastigote-like flagellar cytoskeleton

The initial impression of the *Δift140* cells by light microscopy is that they look like amastigotes as they are shorter and have a much reduced flagellum that does not extend beyond the cell body (figure 1*a–c*). However, the overall shape of *Δift140* cells more closely resembles that of a dividing promastigote than that of an amastigote. Given these changes, we sought to determine which cytoskeletal structures and features had been able to form and what was their configuration. We used a range of techniques that included thin-section transmission electron microscopy (TEM), serial electron tomography and the tagging of endogenous proteins with eYFP to investigate the assembly of different cytoskeletal structures when flagellum elongation was disrupted.

**Figure 3.**
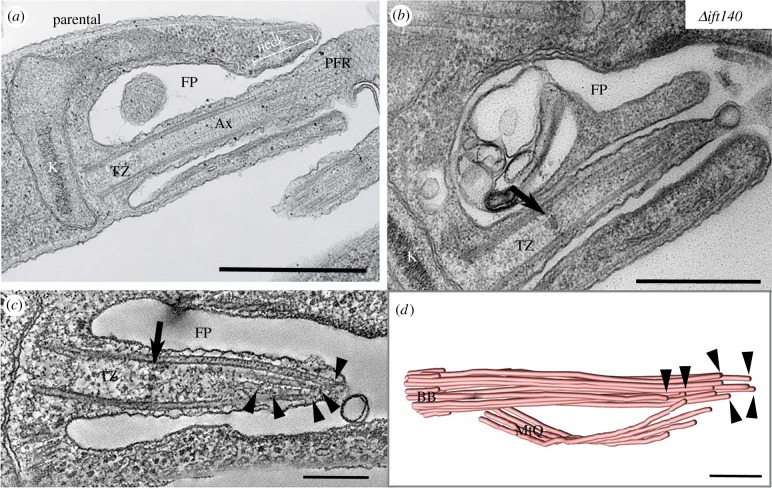
*Δift140* cells have an amastigote-like flagellum. Transmission electron microscopy (TEM) analysis of the anterior region of parental (*a*) and *Δift140* cells (*b–d*). In parental cells, the flagellum has a canonical axoneme (Ax) that elongates from a transition zone (TZ). Starting from the neck region (neck) of the flagellar pocket (FP), a paraflagellar rod (PFR) is also found next to the axoneme. Thin-section TEM image (*b*) and serial electron tomography section (*c*) showing the short flagellum of *Δift140* cells, with a collapsed axoneme and a clear transition zone, as defined by the presence of a basal plate (black arrow). Note the absence of a central pair of microtubules, characterizing a 9 + 0 architecture. (*d*) Three-dimensional model of the flagellar microtubules and microtubule quartet (MtQ) from a serial tomogram of a *Δift140* cell, showing axonemal microtubules terminating at different positions at the distal tip (black arrowheads). K, kinetoplast; BB, basal body. Scale bars, 500 nm (*a,b*); 200 nm (*c,d*).

We focused our analysis on the anterior end of the cell, where the flagellum emerges from the flagellar pocket, in the parental cells. Initially, we examined the overall conformation of the flagellum. Parental promastigotes had the typical anterior end organization of flagellum and flagellar pocket associated structures described previously (figure 3*a* and electronic supplementary material, movie S1) [3,6,11,38], and had a kinetoplast located close to the basal body of the flagellum, which included a transition zone that extended into a canonical 9 + 2 axoneme. A lattice-like extra-axonemal structure termed the paraflagellar rod (PFR) was present alongside the axoneme, as is usual in these promastigote parasites (figure 3*a*). The proximal domain of the flagellum was contained within the flagellar pocket, and the flagellum was attached to the cell body along the neck region of the flagellar pocket (figure 3*a*).

TEM and serial tomography confirmed the light microscopy observations (figure 1) that *Δift140* promastigotes possess no external flagellum (figure 3*b* and electronic supplementary material, movie S2). Nevertheless, these cells had a short, unattached flagellum (approx. 0.6–1.1 µm in length; *n* = 39 images of independent cells) contained within the flagellar pocket, at the anterior end of the cell, and projections of the flagellar membrane were often observed emerging from the flagellum tip, at the flagellar pocket opening (figure 3*b*). We could identify a short flagellum in every flagellar pocket examined, demonstrating that the docking of the basal body to the flagellar pocket membrane occurred in *Δift140* cells. This short flagellum had a transition zone that terminated clearly at a basal plate (figure 3*b,c*). Despite the presence of a clear basal plate in *Δift140* cells, no sections through the basal plate showed a central pair emerging from this structure and running towards the distal end of the flagellum. Thus, our data suggest that the central pair of microtubules is absent from the axoneme of *Δift140* cells. In the short axoneme of these cells, the outer doublets collapsed inwards towards the distal end (figure 3*b–d*), which characterizes a 9 + 0 collapsed architecture. Also, three-dimensional reconstructions from tomography data confirmed that axonemal microtubules terminated at different positions (figure 3*d*), similar to that described for *Leishmania* amastigotes, which have a collapsed 9 + 0 (9v) architecture [39–41].

Curiously, the transition zone of *Δift140* cells was longer than that of parental cells (314 ± 38 nm, versus 268 ± 27 for parental cells; *p* < 0.001). In parental cells, the proximal end of the PFR is found within the flagellar pocket neck (figure 3*a*). However, we found no evidence from either thin-section TEM or serial tomography that the short flagellum of *Δift140* cells had a PFR. Overall, the flagellar skeleton in the *Δift140* cells with the collapsed axonemal structure and lack of a PFR is amastigote-like in configuration. This implicates modulation of IFT as the major factor defining the nature of the flagellum when associated with membrane extension.

### 2.4. Flagellar pocket and associated structures are maintained in *Δift140* cells

**Figure 4.**
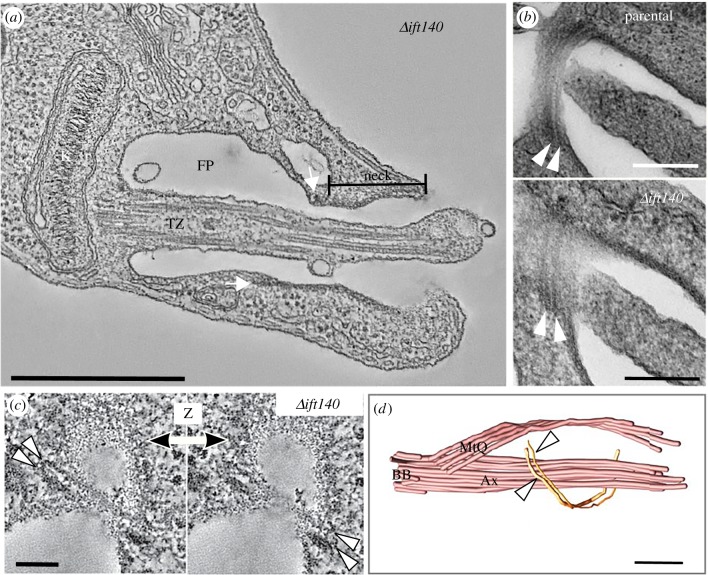
The flagellar pocket structure is preserved after retrograde IFT disruption. (*a*) Section of a serial tomogram of a *Δift140* cell, showing the shape of the flagellar pocket (FP), with the bulbous region near the kinetoplast (K) and an anterior neck region (neck). White arrows indicate the position of the flagellar pocket collar. (*b,c*) Detailed view of the flagellar pocket collar by thin-section TEM (*b*; parental at the top, and *Δift140* at the bottom) and serial tomography (*c*), showing that the two filaments that compose the collar are present in *Δift140* cells (white arrowheads). (*d*) Three-dimensional model from a serial tomogram showing the position of the collar filaments relative to the axoneme (Ax) and the microtubule quartet (MtQ) in a *Δift140* cell. The path of the filaments is interrupted by the microtubule quartet. Scale bars, 500 nm (*a*); 200 nm (*b,d*); 100 nm (*c*).

As observed in parental cells by electron microscopy (figure 3*a* and electronic supplementary material, movie S1), the anterior region of *Δift140* promastigotes was dominated by a flagellar pocket composed of two zones (figure 3*b,c* and electronic supplementary material, movie S2). In the bulbous anterior region, the flagellum was asymmetrically positioned within the pocket lumen. Anterior to the pocket lumen was a roughly cylindrical neck region, where the pocket membrane surrounded the flagellum more closely (figure 4*a*).

The electron microscopy analysis confirmed that *Δift140* cells have a flagellar pocket collar structure similar to that of parental cells, with two collar filaments composed of a thicker proximal filament (relative to the base of the flagellum) and a thinner distal filament—defining the proximal end of the neck region (figure 4*b–d*). The collar is evident in longitudinal sections of the pocket (figure 4*a*) and also in rarer tangential views through the collar region (figure 4*b,c*). We did not observe any differences between the flagellar pocket collar in the *Δift140* cells and the parental cells. To analyse this further, we measured the width of the flagellar pocket collar in TEM images where the collar filaments were present on either side of the neck region. The mean width of the *Δift140* cells was essentially the same as that of the parental cell (489 ± 101 nm versus 498 ± 101 nm, *n* = 30 versus *n* = 10, respectively). In the three-dimensional model of a *Δift140* cell, the collar filaments followed an oblique path relative to the longer axis of the flagellum, forming a C-shaped structure around the collar, with the gap in the C-shape allowing the passage of the microtubule quartet through the collar region (figure 4*d*), as described for wild-type cells [11].

The position of various organelles surrounding the flagellar pocket, such as the Golgi apparatus, has previously been defined based on the axonemal doublet numbering [11]. However, we could not determine axonemal doublet numbering with certainty in tomograms of *Δift140* cells due to the lack of the central pair and of a PFR. A prediction of doublet numbering based on pro-basal body positioning (next to triplets 6–7 of the mature basal body) suggests that the structures surrounding the flagellar pocket and the microtubule quartet (MtQ) nucleation sites were positioned as expected for promastigotes of *L. mexicana* (electronic supplementary material, movie S2) [11].

### 2.5. FAZ filament and microtubule quartet assembly is independent of IFT140

**Figure 5.**
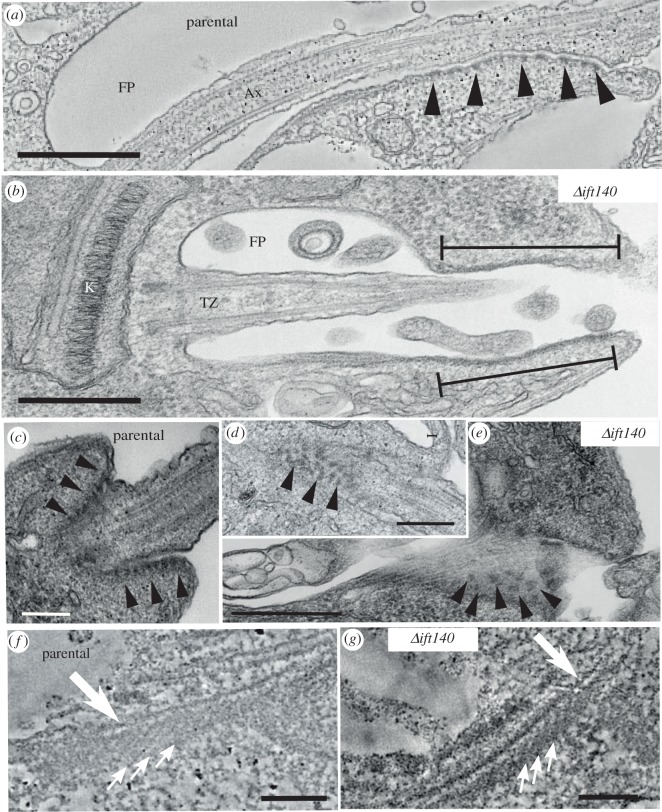
Cytoplasmic FAZ structures are present in *Δift140* cells. (*a*) Parental cells have a clear zone of flagellum attachment to the cell body (viewed here in a serial tomography section), with distinct attachment plaques at the cytoplasmic side of the attachment zone (black arrowheads). (*b*) TEM thin-section of a *Δift140* cell, showing electron-dense areas below the plasma membrane at the neck region, despite the lack of flagellum attachment to the cell (black lines). (*c–e*) Thin-section images showing orderly attachment plaques at the anterior end of parental cells (*c*; black arrowheads), and electron-dense structures likely to correspond to attachment plaques in *Δift140* cells (*d,e*; black arrowheads). (*f–g*) Detailed view of the FAZ filament in parental (*f*) and *Δift140* cells (*g*), in virtual sections of serial tomograms. A conspicuous filament (large white arrow) was observed running alongside a wider striated structure (small white arrows). Scale bars, 500 nm (*a,b,e*); 200 nm (*c,d*); 100 nm (*f,g*).

In addition to the short flagellum, one of the most distinctive abnormalities found at the anterior region of *Δift140* cells was the lack of flagellum attachment to the cell membrane in the neck region, despite the presence of a flagellar pocket similar to that of parental cells (figures 3*b* and 5*b* and electronic supplementary material, movie S2). This suggests that the attachment of the flagellum to the cell body depends on flagellum elongation. Although flagellum attachment to the cell appeared absent, some of the cytoplasmic structures typically found in the cell body domain of the FAZ of *Leishmania* cells [11] were present in *Δift140* promastigotes (figure 5*c–g*; electronic supplementary material, movie S2).

The well-defined electron-dense junctional complexes that represent a classic feature of the FAZ in trypanosomatids (figure 5*a*) [11,42–45], and are particularly prominent at the distal end of the neck in *Leishmania* (figure 5*c*) [11], were less conspicuous in *Δift140* cells (figure 5*b*), in agreement with the lack of flagellum attachment to the neck membrane. However, in *Δift140* cells, we did observe electron-dense material underlying large sections of the cell membrane in the neck region (figure 5*b*). In addition, glancing thin sections of the area underlying the cell membrane at the neck showed electron-dense plaques reminiscent of junctional complexes (figure 5*d,e*), although these were less distinctive and ordered than typical *Leishmania* FAZ junctional complexes (figure 5*a*) [11]. These observations suggest that functional IFT is necessary to establish the overall organization of the FAZ structure.

**Figure 6.**
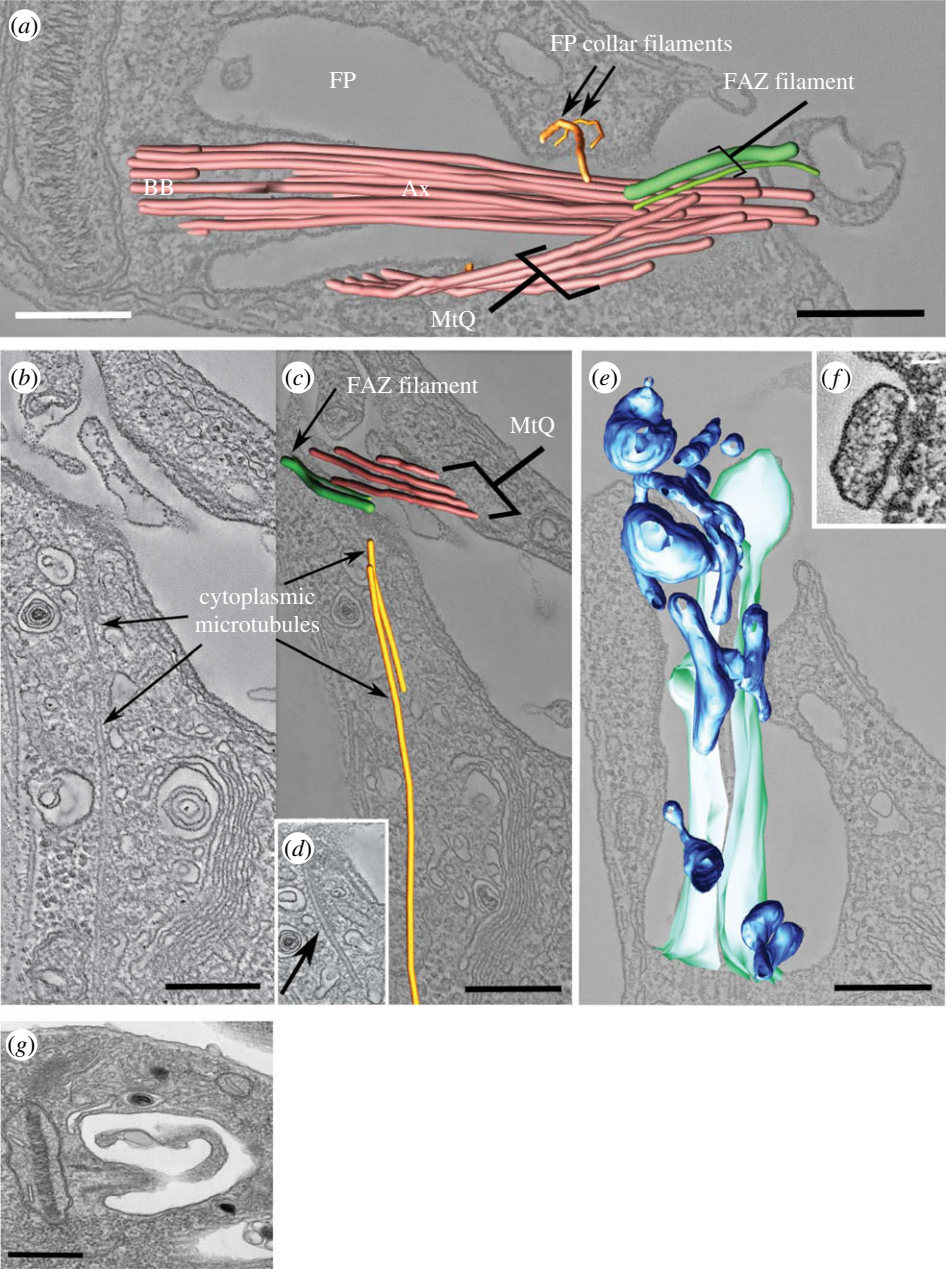
Overall architecture of the structures around and inside the flagellar pocket of *Δift140* cells. (*a*) Three-dimensional model from a serial tomogram, showing the position of the FAZ filament, microtubule quartet (MtQ) and collar filaments relative to the flagellar axoneme (Ax). (*b,c*) Two cytoplasmic microtubules that emerge from the proximal end of the FAZ filament, as seen in tomogram sections (*b,d*) and in three-dimensional models (*c*). While one cytoplasmic microtubule is short (arrow in *d*), the other runs towards the posterior past the Golgi, and beyond the boundary of the tomogram. (*e*) Accumulation of membrane-bound structures (dark blue) in the flagellar pocket lumen. These structures did not appear connected to the flagellar membrane (cyan). (*f*) Membrane-bound structure linked to the flagellar pocket membrane via clear attachment plaques. (*g*) Thin-section TEM image showing membrane being released from the tip of the flagellum. Scale bars, 200 nm (*a–e*); 50 nm (*f*), 500 nm (*g*).

The serial electron tomography analysis showed that the FAZ filament was still assembled in *Δift140* cells (figure 5*g*), showing that FAZ filament assembly was independent of attachment of the flagellum to the cell. The FAZ filament had the same basic structure of that of parental (figure 5*f*) and wild-type cells [11], with a conspicuous filament running alongside a wider structure whose striated nature (with a periodicity of approx. 15 nm) was evident in our tomograms, although it was not described previously. As it crossed the neck region, the FAZ filament was positioned next to a row of electron-dense structures likely corresponding to junctional complexes (figure 5*g*). The tomograms also showed that, despite the lack of flagellum attachment, the microtubule quartet (MtQ) was assembled in *Δift140* cells, following the typical helical path around the flagellar pocket (figures 3*d*, 4*d* and 6*a* and electronic supplementary material, movie S2).

The microtubule cytoskeleton of the trypanosomatid cell body consists largely of an array of subpellicular microtubules below the plasma membrane (and a few flagellar pocket associated microtubules). Nevertheless, a few studies have described a limited number of cytoplasmic microtubules in *T. cruzi* [46,47] and in *L. mexicana* [11,48]. In our tomograms, we detected at least two cytoplasmic microtubules running from the collar region, near the base of the FAZ filament, towards the posterior region of the cell (figure 6*b–d*). The position and length of these microtubules matched those described for wild-type cells [11], with two roughly parallel microtubules, one short (approx. 0.6 µm in length; figure 6*d*) and another considerably longer, running beyond the boundaries of the tomogram (figure 6*c*). The latter microtubule had a path compatible with that of the lysosome-associated microtubule described previously [48]. Overall, the TEM analysis showed that the flagellar pocket and associated structures of *Δift140* cells appeared promastigote-like in configuration, in contrast with the axoneme, which was amastigote-like.

### 2.6. Membranous structures accumulate in the flagellar pocket lumen of *Δift140* cells

A conspicuous feature of the *Δift140* cells was the presence, in the flagellar pocket lumen, of abundant membrane-bound structures of diverse shapes and sizes (large and small vesicles, tubules and multivesicular structures; figure 6*e*), which may correspond to the SMP1::mChFP-positive plasmanemes extending from the anterior end of *Δift140* cells seen by fluorescence microscopy. Interestingly, some of these membrane-bound structures were clearly attached to the membrane of the flagellar pocket neck (figure 6*f*; electronic supplementary material, movie S3), with electron-dense structures resembling junctional complexes found at the cytoplasmic side of the region of attachment, as observed for the attachment region of parental cells. In our thin-section TEM analysis, we observed membranous material extending from the short axoneme with a narrowing of the membrane at the end of the microtubule structure (15 of 25 independent flagellum profiles; figure 6*g* and electronic supplementary material, figure S2A). This material being released from the flagellum could potentially be the source of the SMP1::mChFP-positive plasmanemes and is likely related to the membrane sleeve observed in *T. brucei* IFT RNAi mutants [20].

### 2.7. *IFT140* deletion impacts differentially on particular flagellum attachment zone domains

**Figure 7.**
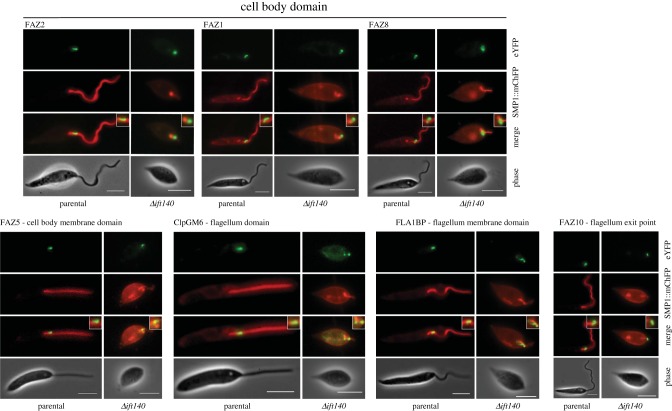
FAZ protein localization is altered in *Δift140* cells. Micrographs of parental and *Δift140* cells expressing different FAZ proteins tagged with eYFP (in green), and SMP1::mChFP (in red). Scale bar, 5 µm.

To further investigate the changes to the FAZ upon *IFT140* deletion, we endogenously tagged seven FAZ proteins with eYFP in *Δift140* cells (figure 7). We chose to tag FAZ proteins that localize to distinct domains of the FAZ, namely: the cell body side of the FAZ, marked by FAZ1, FAZ2 and FAZ8; the cell body FAZ membrane protein FAZ5; and the flagellum side of the FAZ, marked by ClpGM6 and the flagellum FAZ membrane protein FLA1BP [11,15,49,50]. We also examined the localization of FAZ10, a marker for the anterior end of the cell and FAZ, where the flagellum exits the flagellar pocket neck [11].

In *Δift140* cells, the cell body FAZ proteins (FAZ1, FAZ2, FAZ5 and FAZ8) all localized to the FAZ region, but there were subtle differences in the distribution of these proteins between parental and *Δift140* cells (figure 7). In the *Δift140* cells, eYFP::FAZ2 and FAZ5::eYFP appeared as a bright punctate signal in the flagellar pocket region of the cell that often co-localized with an SMP1::mChFP-positive structure, whereas in the parental cell, these proteins were found as a short line at the anterior end of the cell running parallel to the flagellum. In parental cells, eYFP::FAZ1 and FAZ8::eYFP formed a ring around the flagellum with a short line parallel to the flagellum at the anterior end of the cell [11]. In the *Δift140* cells, these proteins still formed a ring across the flagellar pocket region of the cell; however, the signal from the short line appeared to have been reduced to a bright spot (figure 7).

We also observed changes in the localization of the flagellum FAZ domain proteins (ClpGM6 and FLA1BP) in *Δift140* cells. ClpGM6 no longer localized to a small region within the flagellum as the flagellum exited the cell body, but was now found distributed throughout the cytoplasm, and was often concentrated in two spots in the flagellar pocket region of the *Δift140* cell (figure 7). This ‘two-spot’ localization of ClpGM6 in the flagellum is similar to that observed in the *Leishmania* amastigote form [11]. In the parental cells, FLA1BP was found as a discrete, small signal within the flagellum; however, in the *Δift140* cells, FLA1BP had a variable localization pattern often consisting of a number of small spots in and around the flagellar pocket region of the cell with some of these dots of FLA1BP::eYFP signal co-localizing with SMP1::mChFP labelled membrane material (figure 7). Finally, eYFP::FAZ10 localized to the anterior tip of *Δift140* cells, with a localization pattern indistinguishable from that of parental cells (figure 7). Overall, these experiments suggest that the loss of IFT differentially influences the FAZ: the proteins in the cell body FAZ domain reflect a promastigote configuration, while those in the flagellum FAZ domain reflect an amastigote configuration, commensurate with the influence of IFT processes.

## 3. Discussion

Here, we presented a detailed analysis of the effect of *IFT140* deletion on *Leishmania* cell morphogenesis. Our data correlate well with that reported in a previous study showing retrograde IFT ablation in *Leishmania* [32]. We were readily able to generate the *Δift140* mutants—implying that retrograde IFT ablation was not lethal—and the cells had no external flagellum and were therefore immotile. Moreover, in agreement with previous reports, we found that the *Δift140* cells were shorter and shed an abundance of membranous material via the flagellar pocket. The IFT dynein (*LmxDHC2.2*) deletion cells in *L. mexicana* were able to assemble a short flagellum that did not extend beyond the cell body [32], similarly to that observed here for the *L. mexicana Δift140* cells. However, there is a difference between the flagella of *Δift140* cells generated from distinct *Leishmania* species, as the *Δift140* flagellum in *L. donovani* was reported to have no axoneme ([33], S. Beverley 2017, personal communication).

### 3.1. *Δift140* mutant morphology and amastigote differentiation

By cursory inspection, *Δift140* cells appear to have adopted an amastigote configuration to some extent, because our results show that deletion of IFT function is sufficient to produce radical, amastigote-like changes in flagellum organization (from 9 + 2 to a collapsed 9 + 0 (9v)), cell length and flagellum FAZ domain structure. Movement of IFT particles has not been observed in the amastigote flagellum, suggesting that, both during promastigote to amastigote differentiation and in *Δift140* cells, the ‘switching off’ or reduction in IFT activity represents a key step in amastigote flagellum formation [12].

After *IFT140* deletion, the cell body FAZ domain and much of the cytoskeleton and membrane organelle positioning remain more akin to the promastigote configuration as opposed to the amastigote-like configuration of the flagellum and flagellum FAZ domain. Moreover, Adhiambo and colleagues [32] showed that loss of *LmxDHC2.2* did not reduce the expression of the promastigote specific protein LmxMKK or induce the expression of the amastigote specific protein CPB2.8. Therefore, the loss of flagellum elongation does not result in differentiation to the amastigote form. Thus, in a similar manner to the ‘procyclic-to-epimastigote’ structural change in *T. brucei* after ClpGM6 RNAi, it appears that discrete modulation of a cytoskeletal structure in the *Leishmania* flagellum can induce major morphological changes, yet not a full structural conversion, and certainly not a full life cycle stage differentiation [49,51].

### 3.2. Role of flagellum elongation in *Leishmania* morphogenesis

The overall cell layout of the *Δift140* mutant was similar to that of the parental cells, with no defects observed in the positioning and structure of the organelles such as the nucleus and Golgi. The general *Leishmania* cell morphology is, therefore, not dependent on flagellum elongation, and is also independent of attachment of the flagellum at the neck region. The major difference in cell body morphology was the reduction in cell length in the *Δift140* cells, compared with parental cells. It is tempting to speculate that, in *Leishmania*, there is a dependency between flagellum elongation and cell body elongation, as observed in trypanosomes, where flagellum length correlates with cell body length [18,32]. During the *Leishmania* cell cycle, there is an approximate halving and doubling of cell length, with the length reduction occurring before cell division and the length increase observed after division [52,53]. Given that the flagellum continues to elongate after division [53], the increase in cell body length could be coordinated/dependent on flagellum elongation; hence, without flagellum assembly, the cell body may not be able to elongate, generating the shorter cells observed in the *Δift140* cell line. While the dependence of cell body elongation on flagellum growth in *T. brucei* is likely to act physically through the FAZ [14–16], the FAZ in *Leishmania* is comparatively much shorter. Thus, a possible link between cell body growth and flagellum elongation in *Leishmania* may rely on signalling mechanisms rather than on physical interactions between cell body and flagellum FAZ components. There is also the possibility that that cell motility is important for cell elongation in *Leishmania*, with the cytoskeleton responding to the forces applied during swimming, and the lack of movement of the *Δift140* cells may therefore contribute to the shorter cells observed.

### 3.3. Dependency of cytoskeletal structure assembly

Despite the loss of an IFT component, the *Δift140* cells were still able to extend a very short flagellum, demonstrating that basal body docking was independent of flagellum elongation. The short flagellum, however, was highly defective in its cytoskeletal assembly, having no central pair structure and short outer microtubule doublets that collapsed inwards. The *Δift140* cells were able to construct a transition zone, which terminated at a basal plate; however, the transition zone—as defined by basal plate positioning—was longer in the *Δift140* cells. Mispositioning of the basal plate may be due to the absence of positional clues provided by axonemal components that fail to assemble onto microtubules, in the *Δift140* cells. Moreover, this mutant lacked a recognizable PFR structure, suggesting either that the assembly of the PFR is dependent on correct axoneme construction or that a signal or template indication is transmitted from the cell body through the FAZ to initiate PFR assembly. However, flagella without a corresponding flagellar pocket and FAZ in the trypanosome BILBO1 RNAi mutant were able to assemble a PFR [54] and so, on balance, it is likely that initiation of PFR assembly is dependent on a flagellum-based signal not a cell body one.

In the closely related species *T. brucei*, the depletion of both IFT-A and -B proteins is lethal and changes the shape and orientation of the flagellar pocket [17,18,28]. By contrast, in *Leishmania Δift140* cells we did not observe a dramatic change in flagellar pocket architecture, with the flagellar pocket neck and the bulb domains still present and clearly demarcated by the flagellar pocket collar. Thus, our data demonstrate that flagellar pocket formation is not dependent on flagellum elongation, in *Leishmania*.

The short flagellum of *Δift140* cells was not attached to the side of the flagellar pocket neck; however, despite this lack of attachment, the localization of the cell body FAZ proteins was remarkably similar to that observed in the parental cells [11]. Furthermore, the FAZ filament and associated structures such as the MtQ also appeared correctly positioned, as viewed by electron microscopy tomography. This suggests that the initial assembly of the FAZ structures in *Leishmania* is largely independent of IFT. However, the assembly of a correctly structured and organized FAZ is dependent on flagellum elongation, which is reflected by the relatively disorganized nature of the FAZ junctional complexes and the fact that the flagellum FAZ proteins ClpGM6 and FLA1BP were no longer restricted to the FAZ domain within the flagellum of the *Δift140* cells. The lack of flagellum attachment could impact on flagellum FAZ formation directly, by affecting the incorporation of FAZ components within the flagellum, or indirectly, by disrupting axoneme/PFR assembly. Moreover, these experiments provide insights into the dependency relationships within the FAZ itself. Assembly of the cell body and cell body membrane FAZ domains appeared to be independent of the flagellum FAZ domain, as these cell body domains were still able to assemble when there were defects in the formation of the flagellum domain.

There were discrete, individual vesicles observed by EM that were attached to the cell body within the flagellar pocket, via junctional complexes similar to those observed in the FAZ. These attached vesicles may correspond to the bright SMP1::mChFP spots that co-localize with the flagellum FAZ protein FLA1BP. The phenomenon of cytoskeleton-free flagellar membrane remaining attached to the cell via the FAZ is reminiscent of the membrane ‘sleeve’ that forms in trypanosome IFT RNAi cells [20,28].

### 3.4. Flagellar membrane synthesis and delivery is independent of flagellum elongation

The nature of the abundant membranous structures found inside the flagellar pocket of *Δift140* cells is not entirely clear; however, the presence of SMP1 in these structures suggests that they originate from the flagellum itself. Vesicles can be shed from the *Leishmania* flagellum [55], and the abundance of membranous structures in the flagellar pocket is likely due to continued flagellar membrane production in the absence of axoneme elongation, which would lead to the accumulation of flagellar membranes not connected with an underlying flagellar cytoskeleton. This disconnect between membrane production and flagellum cytoskeleton production mirrors the phenomenon with the IFT mutants in trypanosomes, which lead to the formation of a flagellar membrane ‘sleeve’ [20,28]. Is release of the membrane a general phenomenon in flagellum biology? Is flagellar membrane always produced and delivered so that when the flagellum has reached the ‘correct’ length the excess material is released as extracellular vesicles? Moreover, during promastigote to amastigote differentiation, the *Leishmania* cell has to disassemble the flagellum, including most of the flagellar membrane. The loss of vesicular material from the flagellum tip would be a very quick and efficient way of disposing of the excess flagellar membrane [56]. In fact, videomicroscopy data from our laboratory show that, when promastigote cells differentiate into amastigotes inside host macrophages, the shrinking flagellum releases SMP1-labelled strands morphologically compatible with plasmanemes (see electronic supplementary material, movie S1) [12]. Therefore, the release of material observed in the *Δift140* cells may reflect a natural process that occurs during the promastigote to amastigote differentiation. However, while the regressing flagellum during differentiation is external and, thus, is expected to release membranous material mostly into the extracellular environment, the flagellum of *Δift140* cells is contained within the flagellar pocket. Therefore, most excess flagellar membrane shed by the flagellum of *Δift140* cells is expected to accumulate inside the flagellar pocket, which is compatible with our observation that the pocket of these cells contains abundant membranous material (figures 3*b*, 5*b* and 6*e*).

### 3.5. Accumulation of IFT machinery at the flagellum tip

Both IFT140 and the cytoplasmic dynein-2 are part of the IFT-A complex and are therefore involved in the movement of IFT components from the tip to the base of flagella, for recycling. A common observation in IFT-A/cytoplasmic dynein-2 deletion or depletion mutants in other organisms is the accumulation of proteins, including IFT-B components, in a ‘bulge’ at the tip of the flagellum/cilium [28,30,31,57]. It is likely that this lack of recycling of the IFT machinery to the base of the flagellum results in a stoichiometric imbalance of material reaching the flagellum tip, which interferes with flagellum assembly. This recycling defect offers an explanation for the shorter cilia/flagella found in IFT-A or cytoplasmic dynein-2 deletion/depletion mutants in many (albeit not all) organisms [28,30,31], as shown here for the *Δift140* mutant in *Leishmania*. Although accumulation of IFT52::eGFP at the tip of the flagellum was not clear in most *L. mexicana Δift140* cells, some (relatively minor) bulging was apparent in one of our two serial electron tomograms of a *Δift140* cell. Thus, it appears likely that some tip accumulation of non-recycled material occurs upon IFT-A ablation in *L. mexicana*. In our electron microscopy imaging, membranous material can be observed being released from the tip of the short flagellum, and it is possible that, as recycling is impaired in this mutant, axonemal proteins including IFT52 will be incorporated into plasmanemes as we observed in figure 2*c*, which might account for the reduced tip accumulation of IFT52 in comparison to other organisms.

The loss of flagellum elongation is well tolerated by the *Leishmania* promastigote cell, which likely reflects the modular nature of kinetoplastid cell organization, where a cell is constructed from discrete units that can be assembled in different ways to produce different cell morphologies [49], including the amastigote form of *Leishmania*, which is perfectly viable despite the lack of an external flagellum. In this context, the IFT140 mutant has provided new insights into both the hierarchical dependencies of the cytoskeletal structures in and around the flagellar pocket of kinetoplastids and potential mechanisms for the generation of the amastigote flagellum.

## 4. Material and methods

### 4.1. Cell culture

*Leishmania mexicana* (WHO strain MNYC/BZ/62/M397) promastigotes were grown at 28°C in M199 medium with Earle's salts, l-glutamine, 10% fetal calf serum, 5 mM HEPES-NaOH (pH 7.4), 26 mM NaHCO_3_ and 5 µg ml^−1^ haemin. Cells were maintained in logarithmic growth (between 1 × 10^5^ and 1 × 10^7^ cells ml^−1^) by regular subculture and cell densities were measured with a CASY model TT cell counter.

### 4.2. Generation of *Δift140* cells and FAZ protein tagging

The deletion constructs were generated using fusion PCR as described previously [58]. Briefly, 500 bp regions of the 5′- and 3′-UTRs directly upstream and downstream, respectively, of the *IFT140* gene were amplified by PCR and then combined with either the hygromycin resistance gene or the bleomycin resistance gene in a second round of PCR, to generate PCR products that had the *IFT140* 5′- and 3′-UTR flanking each resistance marker. For the eYFP tagging of the *L. mexicana* FAZ protein and IFT52 homologues, the corresponding ORFs and UTRs were cloned into the pLEnTv2-YB plasmid, as previously described [11,58]. The plasmids and fusion PCR construct were electroporated as previously described [58] using program X-001 on a Nucleofector 2b.

### 4.3. Light microscopy

All cell lines were examined live. For live cell microscopy, cells were washed three times in PBS, resuspended in PBS with Hoechst 33342 (1 µg ml^−1^), and then 10 µl of the cell suspension was placed on a poly-l-lysine-coated slide. Cells were imaged using either a Leica DM5500B or a Zeiss AxioPlan 2 microscope, controlled by the Micro-manager software, and using a 100× objective and a Neo 5.5 sCMOS camera.

### 4.4. Transmission electron microscopy

For TEM, cells were fixed in the culture medium by the addition of 1 ml of 25% glutaraldehyde to 9 ml of culture (in the exponential phase of growth). Then, cells were immediately centrifuged (at 900*g*, for 5 min), washed in a buffered fixative solution (0.1 M PIPES buffer, pH 7.2, containing 2.5% glutaraldehyde and 4% paraformaldehyde), resuspended in fresh buffer fixative solution and fixed overnight at 4°C. Following fixation, cells were washed five times in 0.1 M PIPES buffer, pH 7.2 (including one 30-min wash in 50 mM glycine in the same buffer) and post-fixed in 1% OsO_4_ in the same buffer at 4°C, for 2 h. After post-fixation, cells were washed five times in deionized H_2_O and then stained *en bloc* with 2% aqueous uranyl acetate overnight, at 4°C. Then, samples were dehydrated in ethanol (30%, 50%, 70%, 95% and three changes of 100%) and embedded in Agar 100 resin. Thin sections were stained with Reynolds' lead citrate, prior to imaging in a Tecnai T12 (FEI), equipped with a 16-MP camera (OneView, Gatan).

### 4.5. Serial electron microscopy tomography

For serial tomography, ribbons containing serial sections of approximately 150 nm were produced from samples prepared for TEM as described above, and sections were stained with Reynolds’ lead citrate prior to imaging at 120 kV, in a Tecnai T12 (FEI), equipped with a 16-MP camera (OneView, Gatan). Each individual tomogram was produced from a total of 240 4K × 4K pixel images (120 tilted images each of 0° and 90° axes, with 1° tilting between images) from the same area in each section, acquired using SerialEM (http://bio3d.colorado.edu/SerialEM/). Individual tomograms were produced using eTOMO (IMOD software package; http://bio3d.colorado.edu/imod/), which was also used to join six to seven consecutive tomograms into serial tomogram volumes. Tri-dimensional models from serial tomograms were produced by manual tracing and segmentation of selected structures using 3dmod (IMOD software package).

## Supplementary Material

PCR confirmation of IFT140 deletion

## Supplementary Material

Supplementary Figure 2

## Data accessibility

This article has no additional data.
