# Supplementary material for: Dependency relationships between IFT-dependent flagellum elongation and cell morphogenesis in Leishmania
Source: Open Biol. 2018 Nov 21;8(11):180124. doi: 10.1098/rsob.180124 (PMC6282073; doi:10.1098/rsob.180124)
Supplement: PCR confirmation of IFT140 deletion [file rsob180124supp1.pdf]

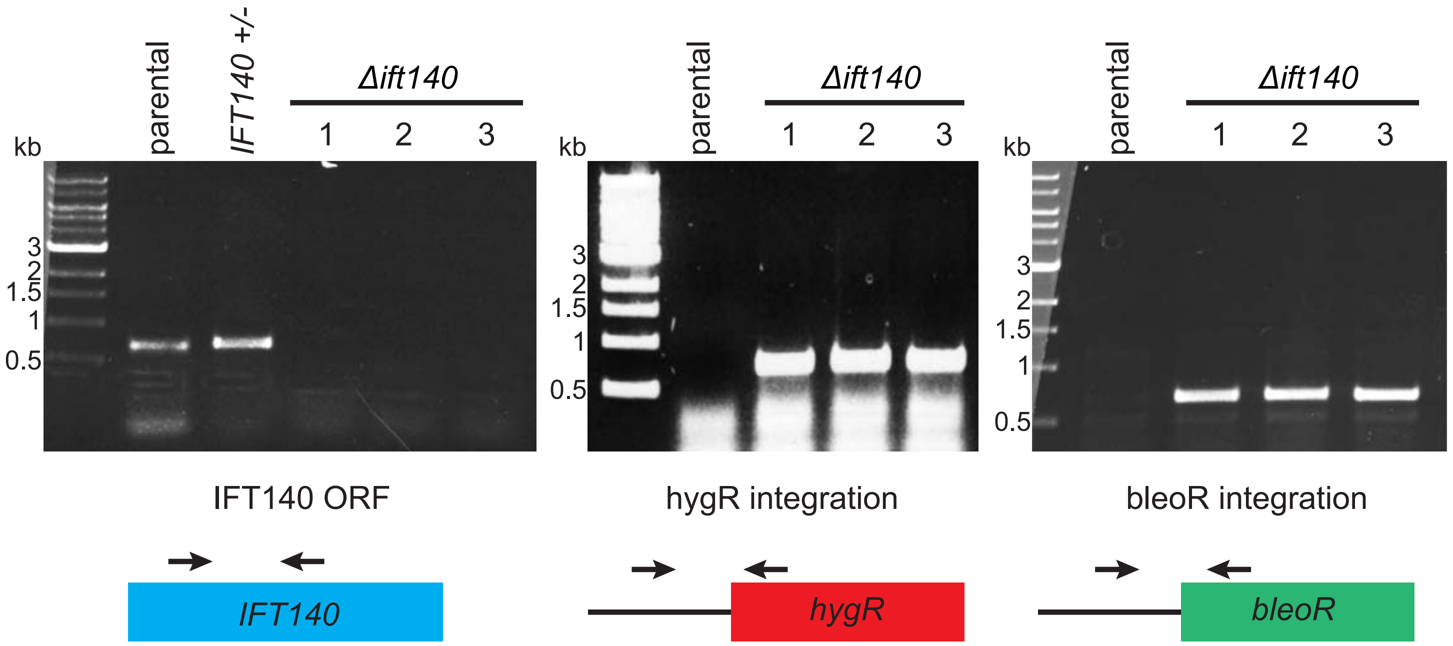

Supplementary Figure 1. PCR confirmation for the loss of *IFT140* gene and integration of resistance markers. Three *Δift140* clones were generated and loss of *IFT140 gene* and correct integration of the resistance marker was confirmed by PCR. The cartoon below the gel image illustrates the region to be amplified by the PCR. Clone 1 was used for all subsequent experimental work.
