# Supplementary material for: Dependency relationships between IFT-dependent flagellum elongation and cell morphogenesis in Leishmania
Source: Open Biol. 2018 Nov 21;8(11):180124. doi: 10.1098/rsob.180124 (PMC6282073; doi:10.1098/rsob.180124)
Supplement: Supplementary Figure 2 [file rsob180124supp2.pdf]

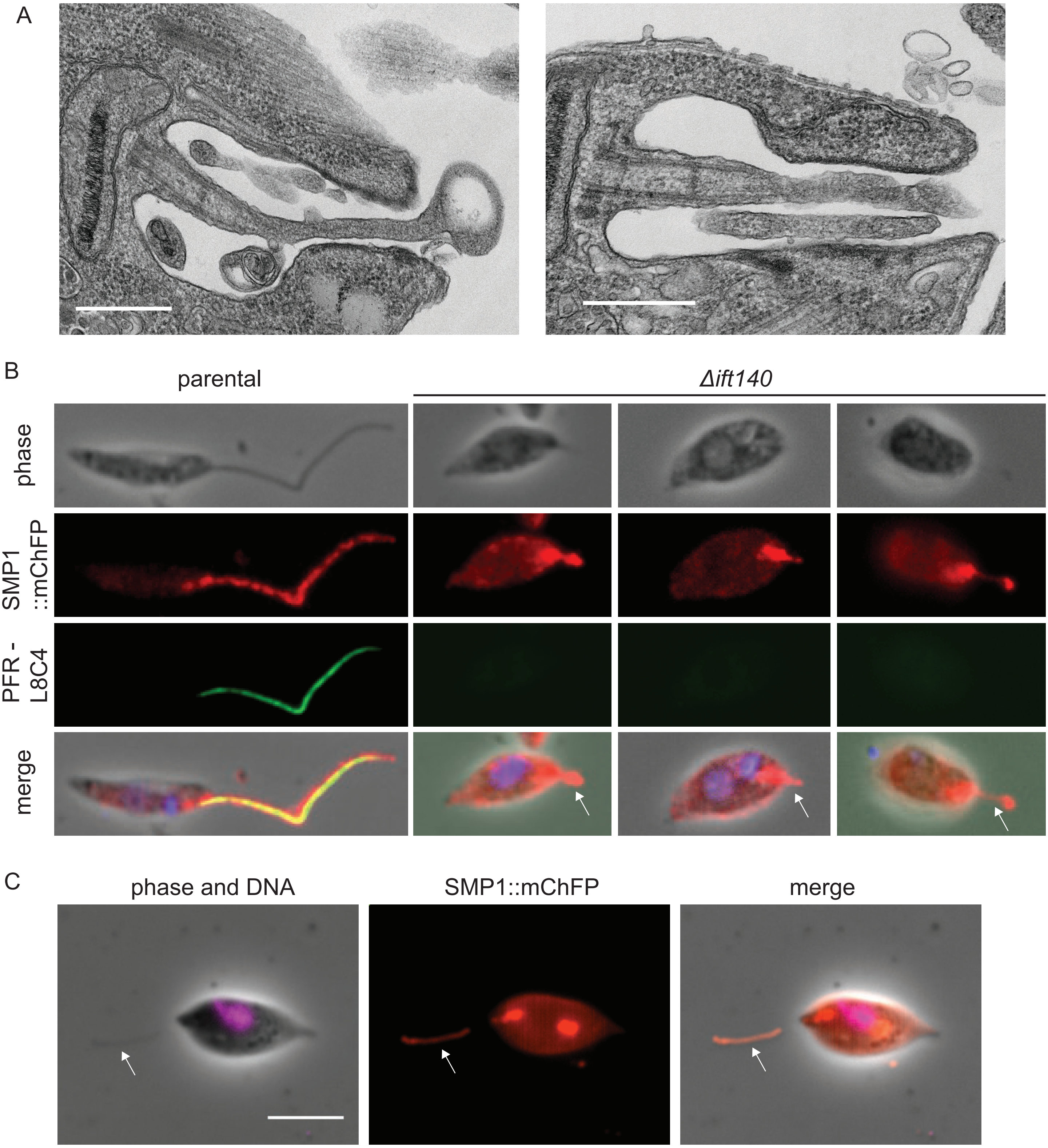

Supplementary Figure 2. Electron microscopy and immunofluorescence of plasmanemes. A) Thin section TEM images of plasmanemes being released from the tip of the flagellum. The plasmanemes do not contain an axoneme or paraflagellar rod. Scale bar is 500 nm. B) Micrographs of methanol fixed parental and *Δift140* cells expressing SMP1::mChFP (red) and stained with L8C4 anti-PFR antibody (green) - PFR is not detectable in plasmanemes (white arrow). Cells were washed 3 times in PBS and settled onto glass slides for 10 minutes. The cells were fixed with methanol for 20 minutes at -20°C and then rehydated in PBS for 30 minutes. Cells were blocked with 10% goat serum in PBS for 1 hour at room temperature and then incubated with L8C4 (1:50) in 10% goat serum in PBS for 1 hour. Slides were washed 4 x 5 minutes in PBS before incubating with the secondary antibody (goat anti-mouse FITC; 1:200) 10% goat serum in PBS for 45 minutes. Slides were washed 2 x 5 minutes in PBS, followed by a 5 minute incubation of PBS with DAPI and then one final 5 minute wash in PBS. Slides were mounted and then imaged. C) Micrographs of live *Δift140* cells expressing SMP1::mChFP (red) with a plasmaneme clearly separated from the cell (white arrow). Scale bar is 5 µm.
